# New Oxidovanadium(IV) Coordination Complex Containing 2-Methylnitrilotriacetate Ligands Induces Cell Cycle Arrest and Autophagy in Human Pancreatic Ductal Adenocarcinoma Cell Lines

**DOI:** 10.3390/ijms20020261

**Published:** 2019-01-10

**Authors:** Szymon Kowalski, Dariusz Wyrzykowski, Stanislaw Hac, Michal Rychlowski, Marek Witold Radomski, Iwona Inkielewicz-Stepniak

**Affiliations:** 1 Department of Medical Chemistry, Medical University of Gdansk, 80-210 Gdansk, Poland; szymon.kowalski@gumed.edu.pl; 2 Faculty of Chemistry, University of Gdansk, 80-309 Gdansk, Poland; daro@chem.univ.gda.pl; 3 Department of General, Endocrine and Transplantation Surgery, Medical University of Gdansk, 80-210 Gdansk, Poland; sthac@gumed.edu.pl; 4 Laboratory of Virus Molecular Biology, Intercollegiate Faculty of Biotechnology, University of Gdansk-Medical University of Gdansk, 80-307 Gdansk, Poland; michal.rychlowski@biotech.ug.edu.pl; 5 Department of Anatomy, Physiology and Pharmacology, University of Saskatchewan, Saskatoon, SK S7N 5E5, Canada; marek.radomski@usask.ca

**Keywords:** Vanadium complex, pancreatic cancer, autophagy, mitotic catastrophe, cell cycle arrest

## Abstract

Pancreatic cancer is characterized by one of the lowest five-year survival rates. In search for new treatments, some studies explored several metal complexes as potential anticancer drugs. Therefore, we investigated three newly synthesized oxidovanadium(IV) complexes with 2-methylnitrilotriacetate (bcma^3−^), *N*-(2-carbamoylethyl)iminodiacetate (ceida^3−^) and *N*-(phosphonomethyl)-iminodiacetate (pmida^4−^) ligands as potential anticancer compounds using pancreatic cancer cell lines. We measured: Cytotoxicity using 3-(4,5-dimethylthiazol-2-yl)-2,5-diphenyltetrazolium bromide (MTT), neutral red (NR) and lactate dehydrogenase (LDH) assay; antiproliferative activity by bromodeoxyuridine BrdU assay; reactive oxygen species (ROS) generation and cell cycle analysis by flow cytometry; protein level by Western blot and cellular morphology by confocal laser scanning microscopy. The results showed that these oxidovanadium(IV) complexes were cytotoxic on pancreatic cancer cells (PANC-1 and MIA PaCa2), but not on non-tumor human immortalized pancreas duct epithelial cells (hTERT-HPNE) over the concentration range of 10–25 μM, following 48 h incubation. Furthermore, molecular mechanisms of cytotoxicity of [4-NH_2_-2-Me(Q)H][VO(bcma)(H_2_O)]2H_2_O (T1) were dependent on antiproliterative activity, increased ROS generation, cell cycle arrest in G2/M phase with simultaneous triggering of the p53/p21 pathway, binucleation, and induction of autophagy. Our study indicates that oxidovanadium(IV) coordination complexes containing 2-methylnitrilotriacetate ligand are good candidates for preclinical development of novel anticancer drugs targeting pancreatic cancer.

## 1. Introduction

While pancreatic cancer is a relatively rare type of cancer, it is the fourth leading cause of cancer-related death worldwide [1,2,3,4]. Pancreatic ductal adenocarcinoma (PDAC) derives from the ductal epithelium of pancreas and constitutes 90% of pancreatic cancers [1]. The lack of specific urine or blood markers, which could be used to identify patients at increased risk, results in delayed diagnosis [5,6]. Consequently, more than 80% of patients are diagnosed with metastatic pancreatic cancer, and at this stage, surgical resection is not recommended [5,6]. The current chemotherapeutic options are, unfortunately, minimally effective. There are two recommended therapeutic strategies gemcitabine and FOLFIRINOX. The latter consists of four compounds: oxaliplatin, irinotecan, 5-flurouracil, and folinic acid [7]. While FOLFIRINOX offers a better survival rate than gemcitabine, this treatment is associated with a significant toxicity and decreased quality of life [7].

Autophagy plays a complex role in the development of cancer [8,9]. Studies have shown that autophagy may increase survival of PDAC cells when exposed to nutrient deprivation, chemotherapy, and hypoxia [10]. However, induction of autophagy may cause pancreatic cancer cell death via apoptosis or autophagy [11,12]. For example, hydroxychloroquine, an inhibitor of autophagy, has now been used in phase II clinical investigation [13].

Growing evidence indicates that metals are essential for the structure and function of many enzymes and biomolecules [14]. For example, metals are involved in electrolyte balance, oxygen transport, electron transfer, and catalysis [14]. Therefore, metal-based anticancer agents, such as cisplatin, have been developed [15]. Metal complexes form new shapes that can more effectively explore new chemical entity (NCE) structure-activity relationship, for example, an octahedral geometry, which better fits the active site of protein kinases [16]. Therefore, inorganic chemistry can exploit the unique properties of metal ions for rational design of NCE and allow for detailed pharmacodynamics, pharmacokinetics, and toxicological investigations [17,18]. Moreover, functionalization of metal complexes with tailor-made ligands can improve the pharmacological profile of NCE as anticancer drugs [19]. Numerous studies have confirmed anticancer potential of metal complexes [20]. Cisplatin was the first metal-based drug approved by the FDA for cancer treatment [21]. Moreover, a derivative of cisplatin, carboplatin, which is used to treat a number of forms of cancer, is less toxic than the parent compound [22]. Recently, the next generation of platinum anticancer drugs have been designed, including nucleotide analogues, nonclassical platinum(II) complexes with trans geometry, or with a mono-functional coordination mode, and a nanoparticle-based drug delivery system designed to deliver platinum(IV) complexes [23,24,25].

Vanadium complexes, in particular organic derivatives, are another metal-based compounds showing anticancer potential [26]. Previously, we have demonstrated that vanadium complexes decrease viability of cancer cells and the molecular mechanism of action may be dependent on organic cations [27]. Therefore, we have synthesized new oxidovanadium(IV) complexes and characterized their pharmacological profile in vitro using cancer and non-cancer human pancreatic cell lines.

## 2. Results

**Figure 1:**
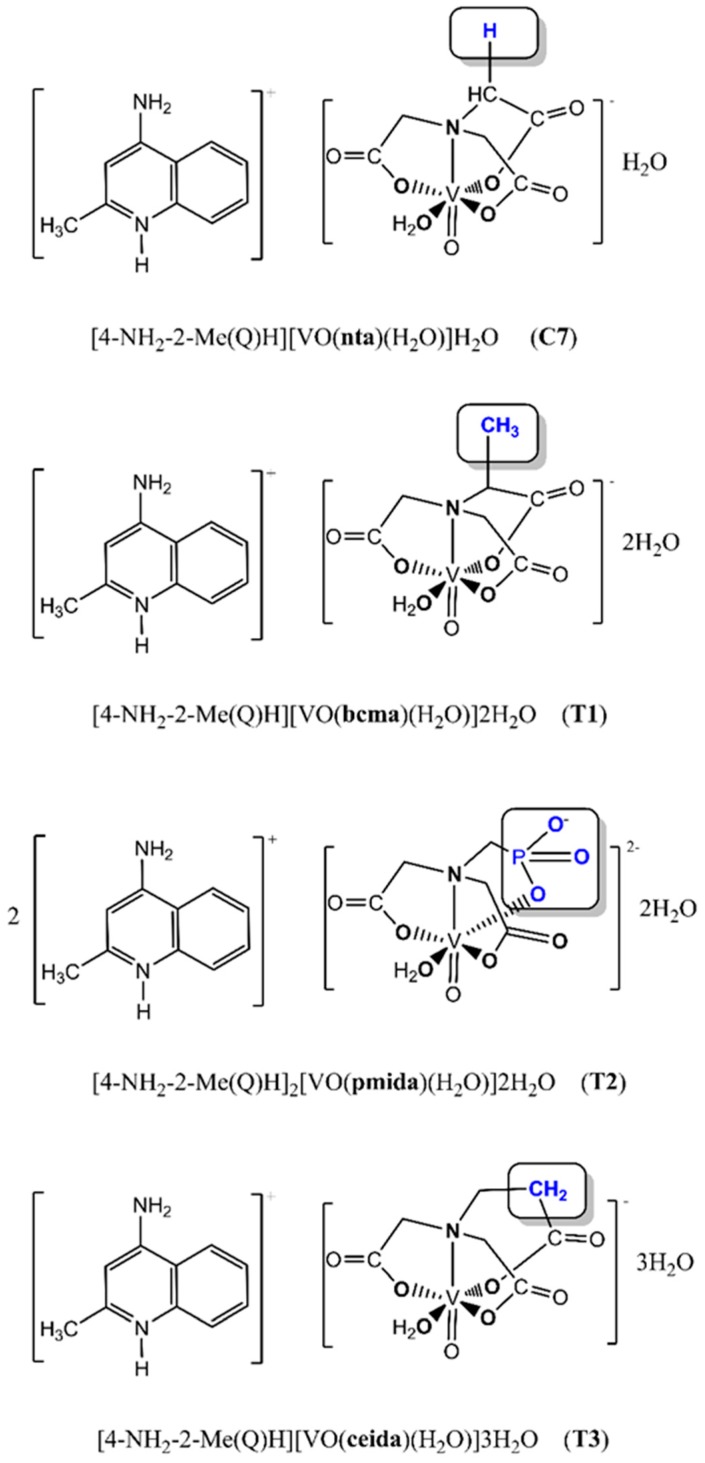
The chemical structures of T1–T3 complexes (derivatives of C7).

We have investigated 3 oxidovanadium(IV) complexes (T1–T3), which are derivatives of C7 (Figure 1), as described by our team [27]. C7 contains the nitrilotriacetate ligand (nta^3−^) and the 4-amino-2-methylquinolinium cation, [4-NH_2_-2-Me(Q)H]^+^ and is cytotoxicity on pancreatic cancer cells through G2/M cell cycle arrest and mitotic catastrophe in the presence of autophagy [27]. In newly synthesized complexes T1–T3, we modified coordination sphere in order to determine and better understand structure-activity relationship. Therefore, we evaluated the cytotoxic profile of vanadium complexes on human pancreas ductal adenocarcinoma cells and compare this profile with immortalized pancreas duct cell line.

### 2.1. Spectroscopic Features of [4-NH_2_-2-Me(Q)H][VO(bcma)(H_2_O)]2H_2_O and [4-NH_2_-2-Me(Q)H][VO(ceida)(H_2_O)]3H_2_O

The characteristic for the oxidovanadium(IV) compounds bands at 971 cm^−1^ ([4-NH_2_-2-Me(Q)H][VO(bcma)(H_2_O)]2H_2_O, Figure S1) and 974 cm^−1^ ([4-NH_2_-2-Me(Q)H]-[VO(ceida)(H_2_O)]3H_2_O, Figure S2) can be assigned to the V=O stretching mode [28,29]. The presence of two bands at 1604 and 1398 cm^−1^ for [4-NH_2_-2-Me(Q)H][VO(bcma)(H_2_O)]2H_2_O and at 1600 and 1395 cm^−1^ for [4-NH_2_-2-Me(Q)H]-[VO(ceida)(H_2_O)]3H_2_O correspond to the antisymmetric and symmetric vibrations of the ionized COO^−^ groups, respectively. This confirms the contribution of the carboxylate groups in the coordination of VO(IV) in a monomeric [VO(bcma)(H_2_O)]^−^ and [VO(ceida)(H_2_O)]^−^ coordination entities. In the IR spectra of the investigated compounds, antisymmetric and symmetric OH stretching, and HOH bending bands of lattice and coordination water are presented at 3300–3100 cm^−1^ and 1660–1610 cm^−1^, respectively. In the same IR range there are bands originated from the antisymmetric (ca. 3415 cm^−1^) and symmetric (ca. 3254 cm^−1^) vibrations of –NH_2_ group. The identification of these bands is, however, difficult due to the presence of absorption bands of OH groups. Indeed, when compared to the corresponding –NH_2_ group bands presented in the IR spectrum of 4-NH_2_-2-Me(Q) (Figure S3), in the IR spectra of the new complexes, the bands are partially masked by the absorption bands of the OH groups.

### 2.2. Magnetic Properties

The effective magnetic moment values were calculated from the equation:

μeff = 2.83 (χm ∙ T)^1/2^

where μeff is the effective magnetic moment, χm is the magnetic susceptibility per one V(IV) center, and T is the absolute temperature.

Plots of the reciprocal magnetic susceptibility, χm and χmT product versus T (χm is the molar magnetic susceptibility for one V(IV) ion) are shown in Figures S4 and S5. At room temperature, the χmT product of the investigated compounds is equal to 0.203 cm^3^·mol^−1^·K (1.27 B.M.) and 0.283 cm^3^·mol^−1^·K (1.50 B.M.), respectively, for [4-NH_2_-2-Me(Q)H][VO(bcma)(H_2_O)]2H_2_O and [4-NH_2_-2-Me(Q)H][VO(ceida)(H_2_O)]3H_2_O.

The value of product χmT slightly increases with temperature lowering and reaches 0.327 cm^3^·mol^−1^·K (1.62 B.M.) for [4-NH_2_-2-Me(Q)H][VO(bcma)(H_2_O)]2H_2_O (Figure S4) and 0.355 cm^3^·mol^−1^·K (1.69 B.M.) for [4-NH_2_-2-Me(Q)H][VO(ceida)(H_2_O)]3H_2_O (Figure S5) at 4 K, which corresponds to a single electron of the 3d1 system [30,31]. The magnetic susceptibility data indicate the absence of magnetic exchange in [4-NH_2_-2-Me(Q)H][VO(bcma)(H_2_O)]2H_2_O and [4-NH_2_-2-Me(Q)H][VO(ceida)(H_2_O)]3H_2_O (Figures S4 and S5) and confirm the presence of the mononuclear [VO(bcma)(H_2_O)] and [VO(ceida)(H_2_O)] coordination units in the structure of the tested compounds (T1 and T3).

### 2.3. The Stability of the Complexes in Aqueous Solutions

The values of stability constants of the investigated complexes were calculated using the equilibrium model presented in Table 1. Diagrams showing species distribution as a function of pH were obtained using the HySS program [32] and are presented in Figures S6–S8.

The [VO(bcma)(H_2_O)]^−^, [VO(pmida)(H_2_O)]^2−^ and [VO(ceida)(H_2_O)]^−^ complexes predominate in the 2.5–6 pH range. Above pH 6, the complexes undergo hydrolysis and the resulting hydroxo complex species ([VO(bcma)(OH)]^2−^, [VO(pmida)(OH)]^3−^, and [VO(ceida)(OH)]^2−^) dominate in the solution (pH > 8).

At the physiological pH (around pH 7.4), the oxidovanadium(IV) complex anions exist in the solution as [VO(bcma)(H_2_O)]^−^ (77%) and [VO(bcma)(OH)]^2−^ (23%), [VO(pmida)(H_2_O)]^2−^ (83%), and [VO(pmida)(OH)]^3−^ (17%), as well as [VO(ceida)(H_2_O)]^−^ (96%) and [VO(ceida)(OH)]^2−^ (4%).

The stability of the [VO(bcma)(H_2_O)]^−^ and [VO(ceida)(H_2_O)]^−^ complexes is similar (Table 1). In contrast, [VO(pmida)(H_2_O)]^2−^ is more stable than the corresponding 2-methylnitrilotriacetate and *N*-(2-carbamoylethyl)iminodiacetateoxidovanadium(IV) complexes. This phenomenon can be explained by the differences in the basicities of the functional groups engaged in the coordination of the VO^2+^ cation. Substitution of CO_2_^−^ by PO_3_^2−^ increases the stability of the [VO(pmida)(H_2_O)]^2−^ complex due to the higher basicities of the phosphonic functions. This is in agreement with a generally observed phenomenon of increased stability of complexes with increased basicity of a ligand [33,34].

### 2.4. Cytotoxic Activity of New T1-T3 Complexes on Pancreatic Cancer Cells in Comparison with C7 Complex

**Figure 2:**
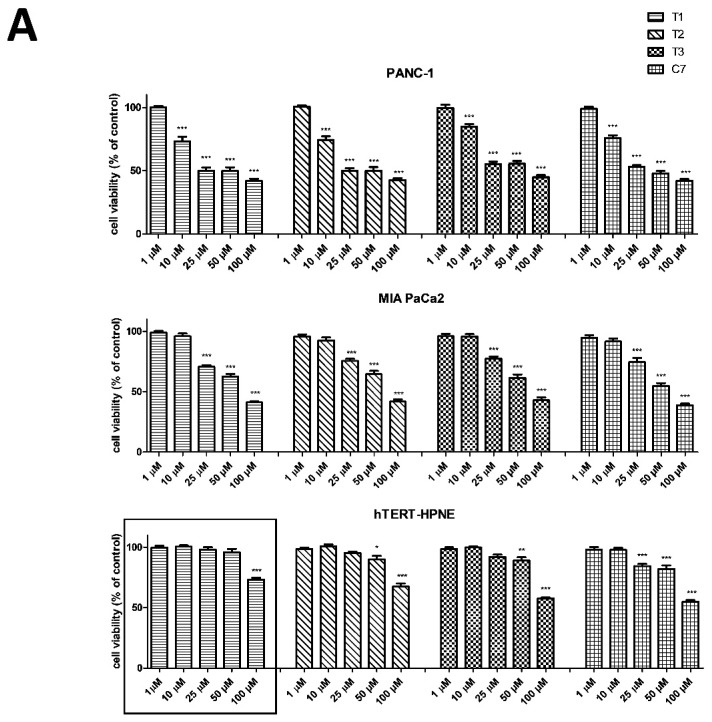

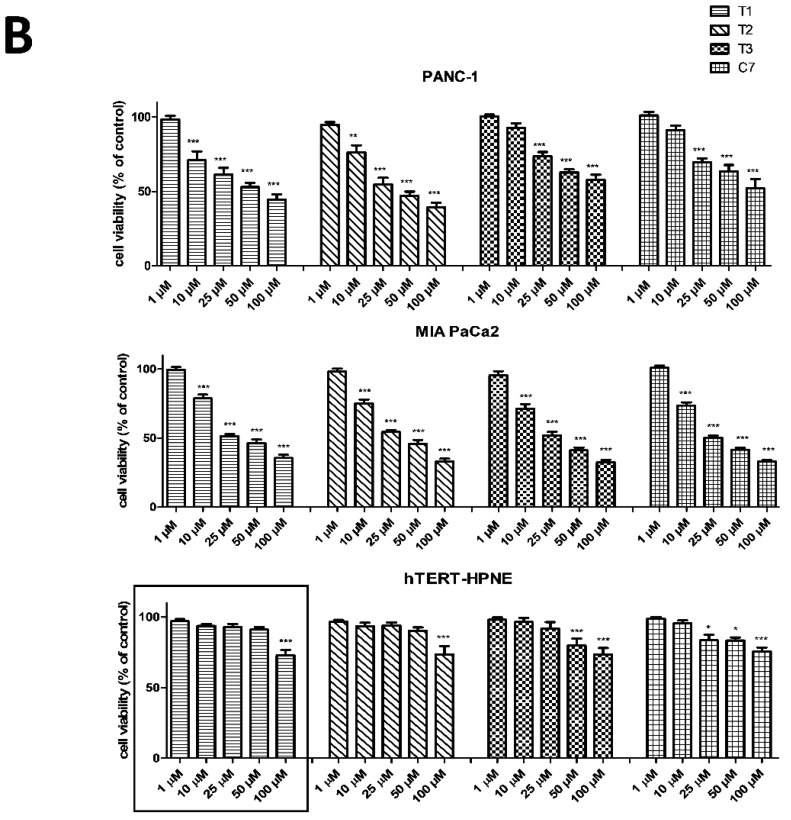
Cytotoxicity of vanadium complexes on pancreas ductal adenocarcinoma cell lines (PANC-1, MIA PaCa2) and immortalized pancreas duct cells (hTERT-HPNE). Cytotoxicity was measured as cell viability using MTT (**A**) and NR (**B**) assays. Cells were exposed to four vanadium complexes (T1-T3 and C7 for comparison) at concentrations ranging from 1 to 100 µM for 48 h. The marked results were shown the least cytotoxic effect on hTERT-PHNE cell line. Data are mean ± SD of 3 separate determinations. * *p* < 0.05; ** *p* < 0.01; *** *p* < 0.001, as compared with untreated cells.

We used the (MTT) and (NR) assays to evaluate the cytotoxicity of four vanadium complexes T1–T3 and control C7 at concentrations ranging from 1 to 100 μM on two human pancreas ductal adenocarcinoma cell lines (PANC-1 and MIA PaCa2) and non-tumor immortalized pancreas duct cells (hTERT-HPNE) following 48 h incubation. Figure 2 shows the effects of T1–T3 and C7 on pancreatic cell lines.

As shown in Figure 2A, B T1–T3 decreased cell viability in a concentration-dependent manner. The pharmacological potency of T1–T3 showed similar cytotoxic activity against pancreatic cancer cells, however, they were less cytotoxic to hTERT-HPNE cells compare with C7 after 48 h of exposure according to MTT and NR assay. Moreover, MIA PaCa2 cell line was more resistant to vanadium complexes than PANC-1 cells according to MTT results, while NR assay exactly showed the opposite cytotoxic effect. T1–T3 compounds exhibited selective cytotoxic effects against PANC-1 in the range of 10–25 µM concentration and 25 μM concentration for MIA PaCa2 according to MTT results. Interestingly, T1 complex was shown selective cytotoxicity even at concentration of 50 µM.

As T1, T2, and T3 showed equal potency as cytotoxic agents on cancer cell lines (Table 2), T1 was selected to examine the mechanisms underlying pharmacological effects of this complex.

### 2.5. Effects of T1 on Pancreatic Cell Proliferation

**Figure 3:**
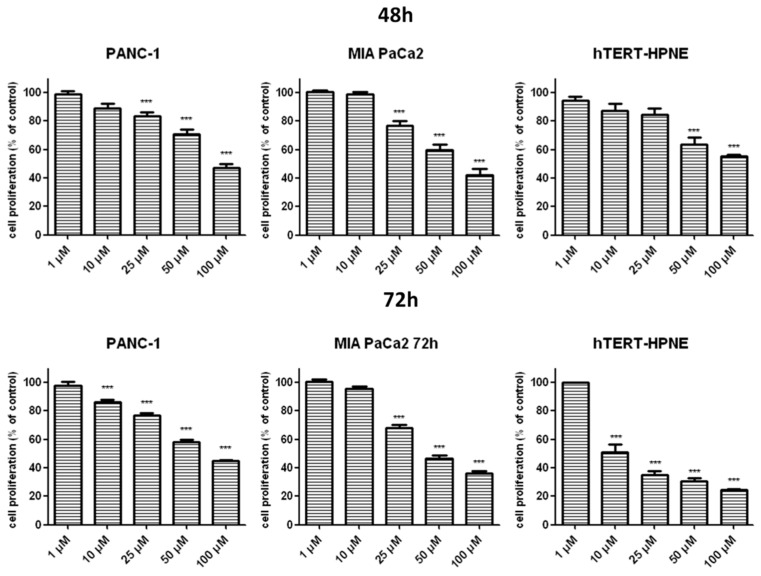
Antiproliferative effects of T1 on pancreas ductal adenocarcinoma cell lines PANC-1, MIA PaCa2 and hTERT-HPNE. Inhibition of PANC-1 and MIA PaCa2 cells proliferation was detected by quantitative ELISA analysis of BrdU incorporation after 48 and 72 h of treatment with vanadium compound. Data are mean ± SD of 3 separate determinations. *** *p* < 0.001, as compared with untreated cells.

Figure 3 indicates that T1 inhibited proliferation of cancer cells in a concentration and time-dependent manner. Of note, cancer cells were less susceptible to the proliferating effects of T1 than hTERT-HPNE cells at 25 μM following 48 h incubation.

### 2.6. Effects of T1 on Necrosis and Apoptosis

**Figure 4:**
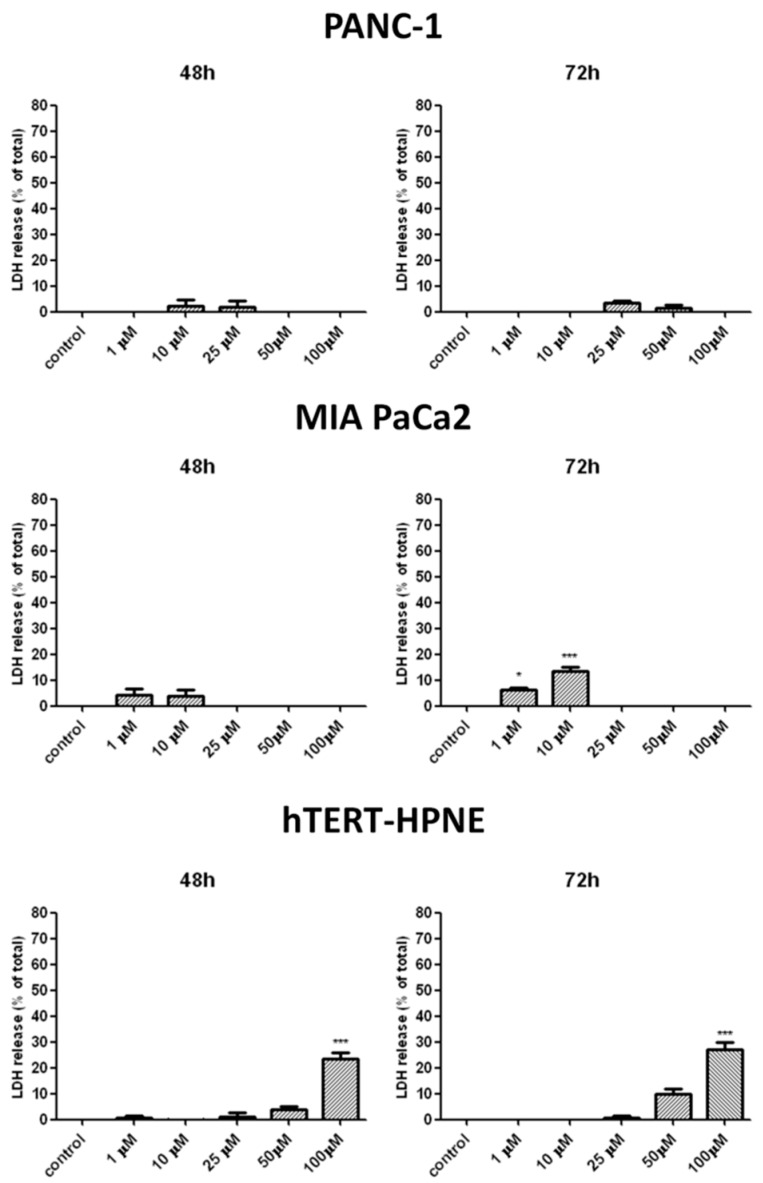
LDH release from PANC-1, MIA PaCa2 and hTERT-HPNE cells after 48 h and 72 h of incubation in the presence of the T1 complex. Data are mean ± SD of 3 separate determinations. * *p* < 0.05; *** *p* < 0.001, as compared with untreated cells.

The release of LDH from pancreatic cells was used to measure the effects of T1 on necrosis and late stage apoptosis [36]. Incubation of pancreatic cancer cells with T1 vanadium complex for 48 and 72 h did not release LDH (Figure 4) from PANC-1 cells. In contrast, T1 caused small, but a significant release of LDH from MIA PaCa2 and hTERT-HPNE cells (Figure 4).

### 2.7. Effects of T1 on ROS Generation

**Figure 5:**
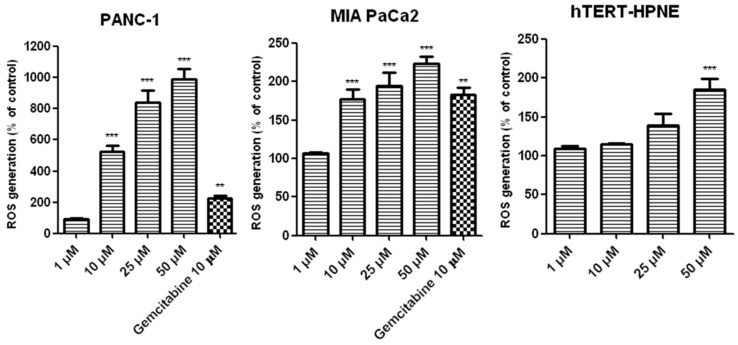
The levels of ROS induced by T1 vanadium complex in PANC-1, MIA PaCa2 and hTERT-HPNE cells following incubation for 48 h. Gemcitabine was used as a positive control. Data are mean ± SD of 3 separate determinations. ** *p* < 0.01; *** *p* < 0.001, as compared with untreated cells.

Figure 5 shows that T1 induced ROS generation in pancreatic cells a concentration-dependent manner. Of note, increased generation of ROS in hTERT-HPNE cells was only detected at 50 μM T1. Gemcitabine, which has been shown to decrease the viability of PANC-1 and MIA-PaCa2 cells through increased generation of ROS [37], used it as a positive control.

### 2.8. Effects of T1 on Cell Cycle in Pancreatic Cells

**Figure 6:**
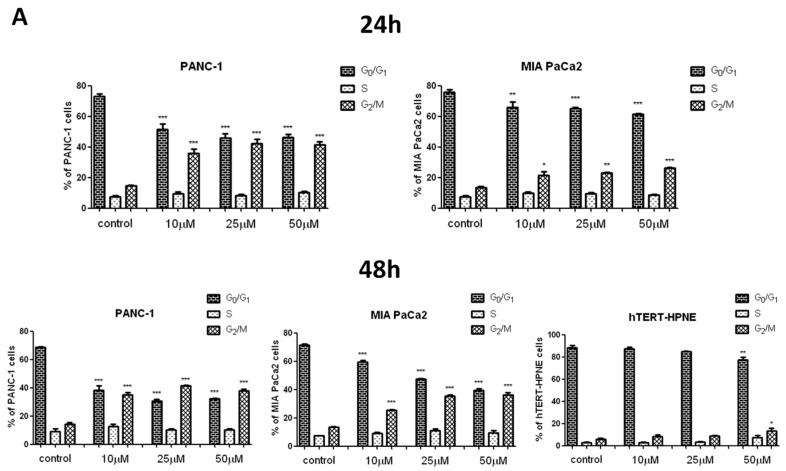

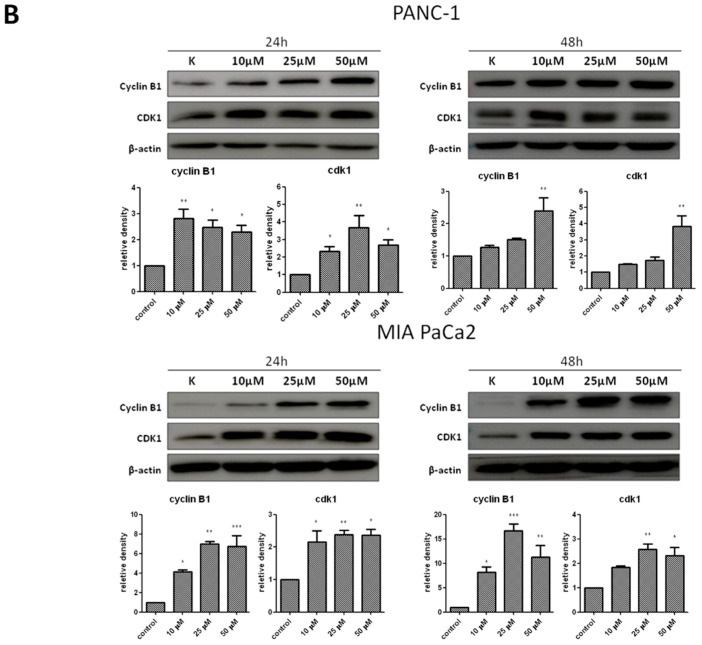
The cell cycle analysis of PANC-1, MIA PaCa2 and hTERT-HPNE cells treated with vanadium complex T1 after 24 h and 48 h of incubation. (**A**) The percentage of cells in each phase. (**B**) Western blot of cyclinB1 and cdk1 expression in cancer cells. Results are given as mean ± SD of 3 separate determinations. * *p* < 0.05; ** *p* < 0.01; *** *p* < 0.001, as compared with untreated cells.

Flow cytometry and Western blot were used to measure the effects of T1 on cell cycle. Figure 6A shows that T1 resulted in G2/M cell cycle arrest in cancer cell lines. In contrast, the arrest in hTERT-HPNE cells was only observed at 50 μM T1. Consistent with these findings, the expression of cyclinB1 and cdk1 proteins in cancer cells was significantly increased after treatment with T1 complex for 24 h and 48 h (Figure 6B).

### 2.9. Effects of T1 on Autophagy and Binucleation in Cancer Cells

**Figure 7:**
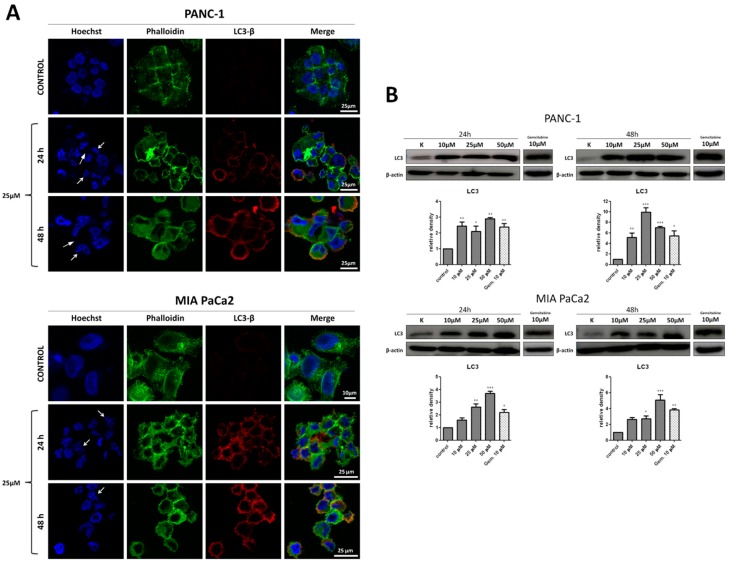
Morphological features and the expression of LC3 protein following incubation of cancer cells with T1. (**A**) Immunocytochemical staining of PANC-1 and MIA PaCa2 cells exposed to T1 (25 µM) for 24 and 48 h. (**B**) Western blot analysis of LC3 expression in PANC-1 and MIA PaCa2 cells exposed to T1 (1–50 μM) for 24 h and 48 h. Gemcitabine was used as a positive control. β-actin was used as an internal control. Results are given as mean ± SD of 3 separate determinations. * *p* < 0.05; ** *p* < 0.01; *** *p* < 0.001, as compared with untreated cells.

Confocal laser scanning microscopy was used to evaluate morphology and autophagy in cancer cells treated with T1 (25 μM). Untreated PANC-1 and MIA PaCa2 cells (Figure 7A) showed morphology typical of adherent cells. The structure of nuclei was disrupted in both cell lines after treatment with T1 for 24 and 48 h. The observed binucleation (Figure 7A white arrows) is indicative of abnormal cell division and this could be a result of mitotic catastrophe [38]. No apoptotic bodies, which are characteristic for apoptosis, were observed [39].

T1 increased autophagy in cells as determined by immunocytochemistry (Figure 7A) and Western blot (Figure 7B) of LC3 protein that is associated with the autophagosomal membrane [40]. Gemcitabine was used as a positive control (Figure 7B) [37].

### 2.10. Effects of T1 on Expression of p53, p21 and RAGE Proteins

**Figure 8:**
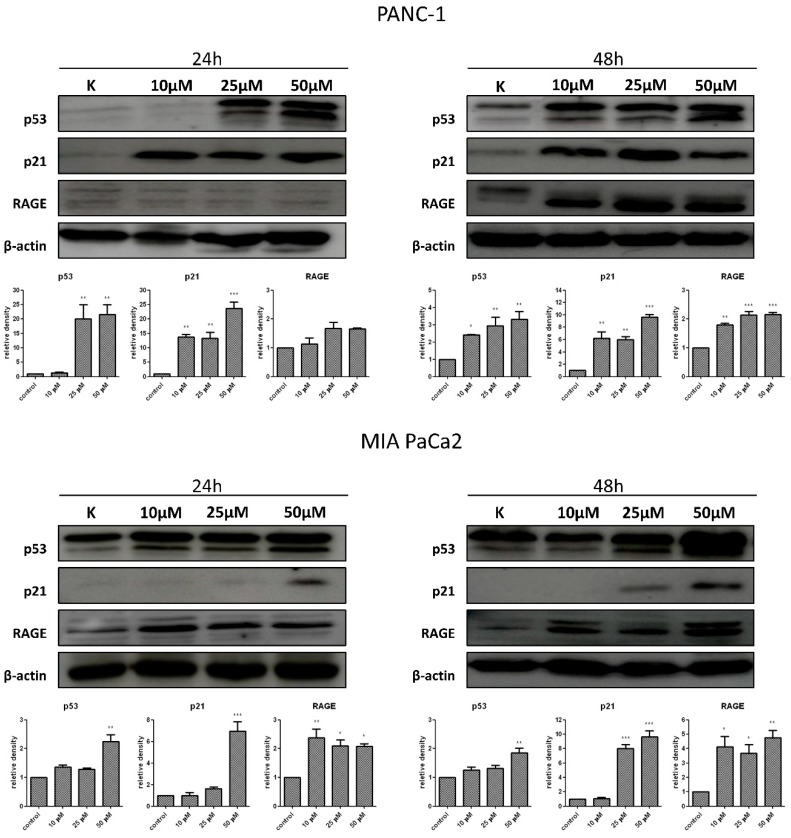
Expression of p53, p21 and RAGE in T1-treated cancer cells as measured by Western blot. β-actin was used as internal control. Data are mean ±SD of 3 separate determinations. * *p* < 0.1; ** *p* < 0.01; *** *p* < 0.001, as compared with untreated cells.

The p53 tumor suppression protein is known to induce p21 protein a nonspecific inhibitor of cyclin-cdk complex that causes cell cycle arrest [41]. The receptor for advanced glycation end products (RAGE) is overexpressed in PDAC and has the ability to reduce apoptosis as well as increase the autophagy process [42]. Therefore, the expression of p53, p21, and RAGE was measured in cancer cells. Figure 8 shows that T1 treatment resulted in time and concentration-dependent increases in p53, p21, and RAGE levels.

## 3. Discussion

A number of studies investigated cytotoxic activity of vanadium complexes on various cancer cell lines including breast cancer, cervical cancer, prostate cancer, colorectal cancer, liver cancer, as well as pancreatic cancer [43,44,45,46]. We have previously demonstrated that organic ligands may determine the molecular mechanism of cytotoxic activity ofoxidovanadium(IV) complexes [27]. Therefore, in this study we examined pharmacological effects of three newly synthesized oxidovanadium(IV) complexes with 2-methylnitrilotriacetate (bcma^3−^), *N*-(2-carbamoylethyl)iminodiacetate (ceida^3−^) and *N*-(phosphonomethyl)-iminodiacetate (pmida^4−^) ligands on human pancreatic ductal adenocarcinoma cell lines (PANC-1 and MIA PaCa2) and a non-tumor immortalized pancreas duct epithelial cells (hTERT-HPNE). Our results indicate that the thus functionalized oxidovanadium(IV) complexes are cytotoxic towards pancreatic cancer cells at concentrations that are not cytotoxic for non-tumor pancreatic cells. Therefore, this chemical strategy holds promise for further development of vanadium complexes as selective chemotherapeutics agents.

We have also explored molecular mechanism(s) of action underlying cytotoxic effects of vanadium complexes.

Generation of ROS plays an important role in anti-cancer activity of vanadium complexes [47]. Indeed, we showed that T1 increased the levels of ROS in pancreatic cancer cells in a concentration-dependent manner. Interestingly, this increase was much higher in PANC-1 cells (5–10 folds) compare with MIA-PaCa2 cells (about 2 folds). Our data are compatible with findings by other groups. For example, Wu et al. [46] also observed that ROS generation is implicated in Bis (acetylacetonato)-oxidovanadium (IV)- and sodium metavanadate-induced pancreatic cancer cell death. Hong et al. [48] demonstrated that cytotoxic activity of vanadium complexes in hepatocarcinoma cells BEL-7402 is mediated through ROS generation, DNA fragmentation, and decrease in mitochondrial membrane potential, which ultimately induce apoptosis. Moreover, Scaleze et al. [49] showed that vanadium complexes induce changes of mitochondrial membrane potential and necrosis as well as apoptosis in ovarian cancer cells. Similarly, another study showed that the pro-apoptotic activity of vanadium (IV) complex in human lung carcinoma cells (A549) depends on intracellular ROS increase [50]. Interestingly, Sinha et al. [51] found that vanadium compounds induce apoptosis in human colorectal carcinoma cells (HCT-116) through mitochondrial outer membrane permeabilization in caspase-independent manner.

Increased levels of ROS in pancreatic cancer cells correlate with the G2/M cell cycle arrest [46]. In keeping with this observation, we found that T1 caused cell cycle arrest in G2/M phase and this effect correlated with expression of cyclinB1 and cdk1. The activation of cdk1/cyclin B initiates the entry of the cells from the G2 to the M phase [52]. Wang et al. [53] found that vanadium salt suppressed the growth of an immortalized hepatic cell line by cell cycle arrest in G2/M phase and inducing apoptosis. Interestingly, Wu et al. [54] found that sodium orthovanadate inhibits autophagy in hepatocellular carcinoma cells, which is associated with G2/M cell cycle arrest. However, it has also been shown that vanadium compounds cause the cell cycle arrest in G1/S phase in human liver cancer cell line [55]. Additionally, Hong et al. [48] determined that vanadium complex inhibits cell growth at the G0/G1 phase in hepatocarcinoma BEL-7402 cells. In human colon cancer cells have been shown that vanadium compounds arrested cells mostly at the S phase [56]. Interestingly, Rozzo et al. [57] indicated that the cell cycle arrest occurs at different phases, depending on the chemical form of vanadium.

We showed that T1 caused the cell cycle arrest in G2/M phase and this effect was associated with nuclear deformities, aberrant mitotic division and binucleation. Interestingly, our previous study found that C7 induced bi as well as multi-nucleation, which is a morphological feature of mitotic catastrophe [27]. We also found that the levels of p53 and p21 were increased following cancer cell exposure to T1. It is known that p53 induces transcription of p21, which blocks transition from the G2 to M phase through binding the CDK1-cyclinB complex and preventing its activation [41,58,59]. Interestingly, the p53/p21 pathway inhibits mitotic catastrophe process and plays a crucial role in senescence regulation [60]. Therefore, our results suggest that T1 exerts cytotoxic effects via a number of molecular mechanisms. Interestingly, Biswal et al. [61] observed chromatin condensation and similar nuclear changes in Hep3B cells after treatment with vanadium complex. Importantly, we did not observe a significant increase of LDH release in cancer cells treated with T1 complexes after 48 h or 72 h, which suggests that necrosis is not induced by T1 [36].

We also studied the role of autophagy in T1-induced cytotoxic effects on cancer pancreatic cells. Autophagy plays a complex role in the development of tumor by both tumor-suppressive and pro-tumorigenic roles [8,9]. For example, it has been shown that increased levels of autophagy in PDAC cells can facilitate their survival under stress conditions such as hypoxia, nutrient deprivation, or chemotherapy [10]. On the other hand, it has been demonstrated in PDAC-derived cell lines that gemcitabine induced autophagy leading to apoptotic cell death. Moreover, the inhibition of autophagy reduces gemcitabine-induced apoptotic cell death [62]. In our study, we found increased levels of LC3-II protein in pancreatic cancer cell lines. The amount of LC3-II, which is conjugated to phosphatidylethanolamine of the autophagosomal membrane, is known to correlate with the number of autophagosomes present in cell, whereas LC3-I is a cytosolic form of LC protein [40]. Moreover, we showed that T1 increased the levels of RAGE protein. Receptor for advanced glycation end-product (RAGE) is a transmembrane receptor, which belongs to the immunoglobulin gene superfamily [42]. It has been shown that RAGE enhances tumor cell survival through increased autophagy and reduced apoptosis in pancreatic tumor cells [43].

In summary, we have synthesized novel oxidovanadium (IV) complexes and examined the effects of these compounds on cancer and non-tumor human pancreatic cell lines. We found that these compounds exerted cytotoxic effects on cancer cell lines, while the effect on non-tumor cell lines was less pronounced. The mechanisms of cytotoxic effects of oxidovanadium (IV) complexes is likely to be dependent on generation of ROS, the cell cycle arrest in G2/M phase with simultaneous activation of the p53/p21 pathway. We suggest that coordination modified oxidovanadium (IV) complexes can be used for further preclinical studies to develop more effective therapeutic strategies for pancreatic cancer.

## 4. Materials and Methods

### 4.1. Synthesis of the Complexes

The synthesis of [4-NH_2_-2-Me(Q)H][VO(bcma)(H_2_O)]2H_2_O (T1) was carried out using a procedure similar to that reported for the preparation of other compounds containing nitrilotriacetateoxidovanadium(IV) moieties [27,63,64]. Thus, the mixture of VO(acac)_2_ (2.65 g, 0.01 mol) and Na_3_bcma (2.71 g, 0.01 mol) in water H_2_O (200 mL) was provided by a rotary evaporation. The mixture was heated under vacuum at 70 °C in order to eliminate Hacac. In the next step, to the obtained concentrated reaction mixture an ethanol solution of 4-amino-2-methylquinoline, 4-NH_2_-2-Me(Q), (0.01 mmol) was added. The compound crystallized directly from this mixture as bluish crystals after approx. 14 days in a refrigerator. By using the same procedure, [4-NH_2_-2-Me(Q)H][VO(ceida)(H_2_O)]3H_2_O was obtained. The synthesis and physicochemical properties of [4-NH_2_-2-Me(Q)H]2[VO(pmida)]3H_2_O have been described previously [65].

All compounds were air-dried at the room temperature and their composition was confirmed by elemental analyses of carbon and hydrogen (Vario EL analyzer Cube CHNS). Anal. Calcd for [4-NH_2_-2-Me(Q)H][VO(bcma)(H_2_O)]2H_2_O: C, 42.29%, H, 5.23%, and N, 8.71%, Found: C, 42.19%, H, 5.22%, and N, 8.65%. Anal. Calcd for [4-NH_2_-2-Me(Q)H]2[VO(pmida)(H_2_O)]2H_2_O: C, 45.30%, H, 5.19%, and N, 10.57%, Found: C, 45.44%, H, 5.25%, and N 10.37%. Anal. Calcd for [4-NH_2_-2-Me(Q)H][VO(ceida)(H_2_O)]3H_2_O: C, 40.83%, H, 5.45%, and N, 8.40%, Found: C, 40.97%, H, 5.43%, and N 8.37%.

Aqueous solutions of the investigated compounds have shown a high stability, e.g., being resistant to the oxidation in air, i.e., remain unaltered (UV–Vis control) for at least 3 days.

### 4.2. The IR Spectra

The IR spectra were recorded on the BRUKER IFS 66 spectrophotometer in a KBr pellet over the 4400–650 cm^−1^ range.

### 4.3. Magnetic Studies

The magnetic measurements of the compounds studied were carried out over the temperature range 4–300 K and at a magnetic induction of 0.1 T using the Quantum Design SQUID-VSM magnetometer (San Diego, CA, USA). The palladium rod sample was used for calibrating the SQUID magnetometer (San Diego, CA, USA). During data analysis the corrections for the sample holder and diamagnetism of the constituent atoms were taken into account [66].

### 4.4. Potentiometric Titrations

All details for the measuring devices and the experimental setup were described in [67]. The electrode was calibrated according to IUPAC recommendations [68]. The composition of the titrand solution used in the experiments was as follows: 2 mM VO^2+^ (VOSO_4_), 2.5 mM Na_3_bcma (5 mM HClO_4_), or 2.5 mM H_3_ceida. Each titration was repeated at least three times in order to check the reproducibility of the data.

The equilibrium constants defined by Equations (1) and (2) were refined using the Hyperquad2008 (ver. 5.2.19) computer program [69]:

pM + qL + rH = MpLqHr(1)

(2) $$\beta \mathrm{pqrs}=\frac{\left[M_{p}L_{q}H_{r}\right]}{{\left[M\right]}^{p}{\left[L\right]}^{q}{\left[H\right]}^{r}}$$

The resulting hydroxo complexes of VO(IV): [VO(OH)]^+^ (log*β*_10-1_ = −5.94) and [{VO(OH)}_2_]^2+^ (log*β*_20-2_ = −6.95) [70] were fixed in the evaluation of the pH-metric titration data.

### 4.5. Antibodies and Reagents

The reagents used for the syntheses were of analytical grade and were used without further purification. They were as follows: VO(acac)2 (≥98%, Sigma-Aldrich, Saint Louis, MO, USA), *N*,*N*-bis(carboxymethyl)-dl-alanine trisodium salt (Na3bcma) (≥90%, Sigma-Aldrich), *N*-(Phosphonomethyl)iminodiacetic acid (H4pmida) (95%, Sigma-Aldrich), *N*-(2-carboxyethyl)iminodiacetic acid (H_3_ceida) (≥98.0%, VWR) and 4-amino-2-methylquinoline (4-NH2-2-Me(Q), ≥98%, Sigma-Aldrich). In a biological study, it was used anti-rabbit secondary antibodies as well as polyclonal anti-p53 and RAGE antibodies from Santa Cruz Biotechnology (Dallas, TX, USA). Polyclonal anti-cdk1, cyclinB1, p21, β-actin and LC3β antibodies were purchased from Abcam (Cambridge, UK). Anti-rabbit antibodies conjugated with Alexa Fluor546 were obtained from Thermo Fischer Scientific (Waltham, MA, USA). 3-(4,5-dimethylthiazol-2-yl)-2,5-diphenyltetrazolium bromide (MTT), neutral red (NR), RNAse A, 2′,7′-dichlorofluorescin diacetate (DCF-DA), phalloidin, hoechst, bovine serum albumin (BSA), paraformaldehyde, gemcitabine, and propidium iodide were purchased from Sigma-Aldrich (Saint Louis, MO, USA). BrdU incorporation ELISA assay from Roche (Grenzach-Wyhlen, Germany) and LDH-Cytotoxicity Assay from Promega (Madison, WI, USA).

### 4.6. Cell Culture

The pancreas ductal adenocarcinoma cell lines (PANC-1 and MIA PaCa2) and immortalized pancreas duct cells (hTERT-HPNE) were obtained from the American Type Culture Collections (ATCC). PANC-1 as well as MIA PaCa2 were cultured in Dulbecco’s Modified Eagle Medium (DMEM) with high glucose concentration (4.5 mg/mL), supplemented with 10% fetal bovine serum (FBS), 100 units/mL penicillin and 100 μg/mL streptomycin. In case of MIA PaCa2, the medium was additionally supplemented with 2.5% horse serum (HS). hTERT-HPNE cells were cultivated in medium composed of three volumes of glucose-free DMEM, one volume of medium M3, 5.5 mM glucose, 2 mM glutamate, 750 ng/mL puromycin, 5 ng/mL EGF, and 5% FBS. Cell cultures were incubated in a humidified atmosphere of 95% air and 5% CO_2_ at 37 °C. All experiments were performed on cells with 70–80% confluence.

### 4.7. Cell Viability Assay

The MTT and the NR assay were used to measure cell viability according to the protocol. Briefly, PANC-1, MIA PaCa2 and hTERT-HPNE cells ware seeded in triplicate on 96-well plates at a density of 12–14 × 10^3^ cells/100 µL. Next day, cells were exposed to vanadium complexes (1–100 µM) dissolved in a serum-free medium for 48 h. After this time, the tetrazolium dye (MTT) or the neutral red (NR) was added to medium for 2 h. The absorbance was measured at 492 nm or 540 nm. The data were expressed as the percentage of untreated cells (control), which was set to 100%.

### 4.8. Cell Proliferation

PANC-1, MIA PaCa2 and hTERT-HPNE cells were seeded in triplicate at concentration of 12–14 × 10^3^ cells per well in 96-well plate. The following day, cells were exposed to vanadium complex in concentration range: 1–100 µM for T1, in serum-free culture condition. After 48 h incubation, the antiproliferative activity was measured by a colorimetric BrdU incorporation, ELISA assay as we described previously [27].

### 4.9. Cell Necrosis

Cell necrosis was measured by fluorometric lactate dehydrogenase (LDH) assay (Promega, Madison, Wisconsin, USA) PANC-1, MIA PaCa2 and hTERT-HPNE cells were seeded in triplicate at a density of 12 × 10^3^–14 × 10^3^ cells per well in a 96-well plate. The following day, the PANC-1 and MIA PaCa2 cells were treated with T1 complex under serum free conditions at the range of concentrations: 1–100 μM for 48 h. Lactate dehydrogenase (LDH) release into the surrounding medium was determined according to the manufacturer’s protocol. Lysis buffer was used as a positive control. LDH data were expressed as a percentage of the total LDH released from cells.

### 4.10. Detection of ROS

The flow cytometry technique was used to determine the generation of intracellular ROS level. PANC-1, MIA PaCa2 and hTERT-HPNE cells were seeded into 6-well plates at a density of 10^6^ cells per plate and the next day, cells were treated by oxidovanadium(IV) complex in concentration of 1, 10, 25, and 50 μM for 24 h as well as 48 h. Analysis was performed according to previously described protocol [27].

### 4.11. Cell Cycle Analysis

The flow cytometry technique was used to perform cell cycle analysis. PANC-1, MIA PaCa2 and hTERT-HPNE cells were seeded into 6-well plates at a density of 10^6^ cells per plate. The next day cells were exposed to oxidovanadium(IV) complex (T1) for 24 h as well as 48 h in selected concentrations (10–50 μM). Analysis was performed according to previous described protocol [27].

### 4.12. Immunofluorescence Staining and Confocal Microscopy

PANC-1 and MIA PaCa2 cells were seeded on coverslips (16 × 16 mm Marienfeld Germany), cultured for one day and incubated with oxidovanadium(IV) complex for 24 h as well as 48 h at 25 μM concentration. On the day of an experiment, cells were fixed in 4% paraformaldehyde for 10 min at room temperature, then permeabilized by adding 0.2% Triton-X 100 for 5 min, and blocked with 5% BSA in PBS for 1 h. Cells were next incubated for overnight with anti-LC3β (1:3000) diluted in 5% BSA in PBS. Then, the cells were washed three times with PBS, and incubated for 1 h with secondary antibodies diluted in 5% BSA (1:5000), including anti-rabbit antibodies conjugated with Alexa Fluor546. Next, the cells were stained with Hoechst (50 μg/mL) for 2 min, and the coverslips were mounted to slides using ProLong Diamond AntifadeMountant (ThermoFisher, Waltham, MA, USA). These specimens were imaged using a confocal laser-scanning microscope (Leica SP8X, Wetzlar, Germany) with a 63× oil immersion lens.

### 4.13. Western Blot Analysis

Western blot analysis was performed in order to determine level of: cdk1, cyclinB1, LC3, p53, p21 and RAGE proteins according to previous described protocol [27]. PANC-1 and MIA PaCa2 cells were seeded into 100 mm petri dishes and cultured until reached about 80–90% confluence. After this, cells were incubated with oxidovanadium(IV) complex (T1) at 10, 25, and 50 μM concentration for 24 h and 48 h. Conditioned media were discharged and attached cells washed with PBS, detached, and after all homogenized. After electrophoresis, proteins were transferred onto nitrocellulose membrane (Protran^®^, Schleicher and SchuellBioScience, Dassel, Germany). β-actin was used as a loading control. Protein levels were quantified using densitometry software (ImageQuant TL 7.0 Software, Pittsburgh, PA, USA).

### 4.14. Statistical Analysis

The obtained data was reported as the mean ± SD for triplicate determination of 3 separate experiments. The results were evaluated by statistical methods using GraphPad PRISM version 5 by the one-way analysis of variance and Tukey’s post-hoc test. Significance was determined at the 5% level (* *p* < 0.05). IC_50_ and logIC_50_ were calculated using the GraphPad Prism 5 program V.5 (GraphPad Software Inc., San Diego, CA, USA) by non-linear regression analysis. Each compound of vanadium was tested at least in triplicate.

## Figures and Tables

**Table 1:** Logarithms of the stability constant values (log*β*_pqr_) of the VO(IV) complexes obtained by adapting the equilibrium model to PT data. The corresponding standard deviations are given in parenthesis.

Species	log*β*_pqr_	[VO(bcma)(H_2_O)]^−^ (T1) (This Work)	[VO(pmida)(H_2_O)]^2−^ (T2) [35]	[VO(ceida)(H_2_O)]^−^ (T3) (This Work)
LH2	log*β*_012_	13.55 ± 0.07	16.04 ± 0.03	13.47 ± 0.07
LH	log*β*_011_	10.50 ± 0.06	10.52 ± 0.02	9.85 ± 0.07
ML	log*β*_110_	12.23 ± 0.02	15.14 ± 0.05	12.36 ± 0.03
MLH_-1_	log*β*_11-1_	4.29 ± 0.16	7.06 ± 0.05	3.54 ± 0.11

**Table 2:** Cytotoxic activity of vanadium complexes on PANC-1, MIA PaCa2 and hTERT-HPNE cell lines after 48 h of treatment. Data are expressed as IC_50_ and logIC_50_ (mean ± SD of 3 separate determinations) and were calculated on the basis of MTT and NR determinations.

	**MTT Assay**					
	**PANC-1**		**MIA PaCa2**		**hTERT-HPNE**	
	**IC_50_** **[μM]**	**LogIC_50_± SD** **[μM]**	**IC_50_** **[μM]**	**logIC_50_ ± SD** **[μM]**	**IC_50_** **[μM]**	**logIC_50_ ± SD** **[μM]**
**T1**	44.67	1.650 ± 0.042	72.22	1.859 ± 0.022	140.9	2.149 ± 0.036
**T2**	45.53	1.658 ± 0.040	77.29	1.888 ± 0.026	138.9	2.143 ± 0.029
**T3**	61.45	1.788 ± 0.039	76.05	1.881 ± 0.025	116.8	2.067 ± 0.018
**C7**	45.94	1.662 ± 0.030	63.82	1.805 ± 0.025	121.8	2.086 ± 0.034
	**NR Assay**					
	**PANC-1**		**MIA PaCa2**		**hTERT-HPNE ^1^**	
	**IC_50_** **[μM]**	**logIC_50_± SD** **[μM]**	**IC_50_** **[μM]**	**logIC_50_ ± SD** **[μM]**	**IC_50_** **[μM]**	**logIC_50_ ± SD** **[μM]**
**T1**	59.73	1.776 ± 0.066	39.46	1.596 ± 0.030	>200	-
**T2**	43.65	1.640 ± 0.049	37.92	1.579 ± 0.029	>200	-
**T3**	123.4	2.091 ± 0.063	32.07	1.506 ± 0.033	>200	-
**C7**	99.72	1.999 ± 0.075	33.12	1.520 ± 0.023	>200	-

^1^ Cell viability decrease was too low to calculate IC_50_; The underlined complex was exhibited the most favorable cytotoxic profile.

## Supplementary Materials

Supplementary Materials can be found at http://www.mdpi.com/1422-0067/20/2/261/s1.
