# Supplementary material for: New Oxidovanadium(IV) Coordination Complex Containing 2-Methylnitrilotriacetate Ligands Induces Cell Cycle Arrest and Autophagy in Human Pancreatic Ductal Adenocarcinoma Cell Lines
Source: Int J Mol Sci. 2019 Jan 10;20(2):261. doi: 10.3390/ijms20020261 (PMC6358955; doi:10.3390/ijms20020261)
Supplement: Supplementary file 1 [file ijms-20-00261-s001.pdf]

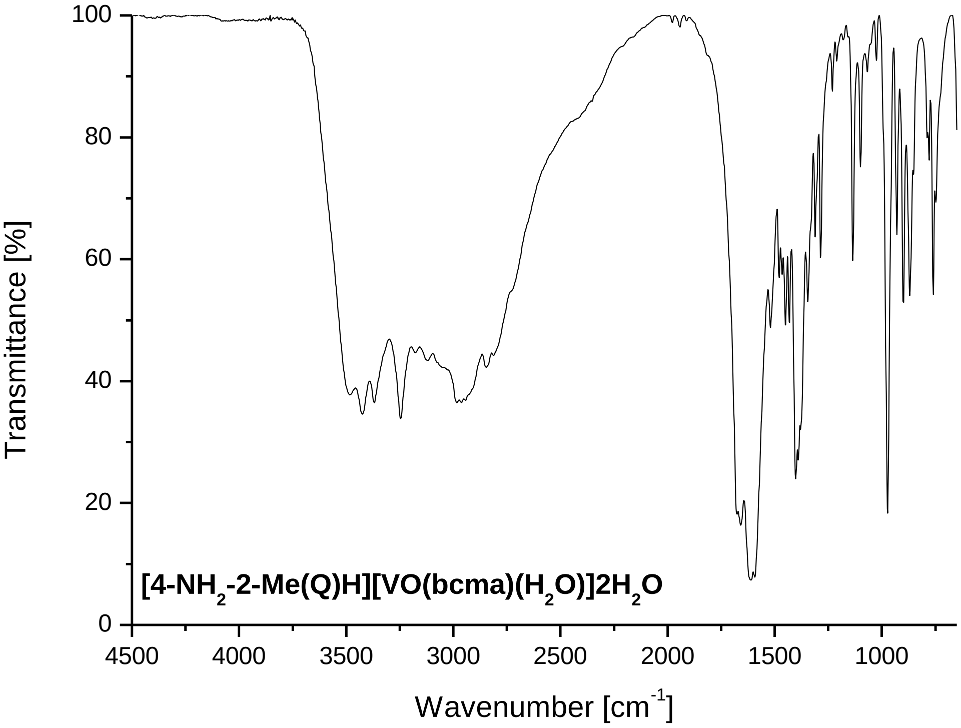

Fig. 1S. The IR spectrum of [4-NH_2-2-Me(Q)H][VO(bcma)(H_2O)]2H_2O.

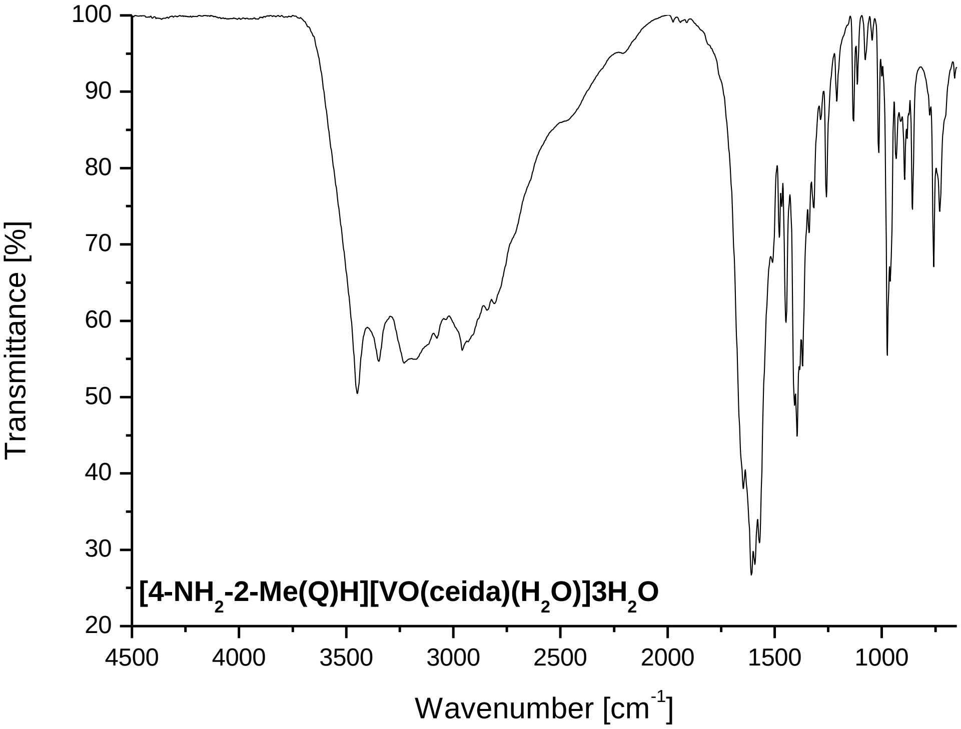

Fig. 2S. The IR spectrum of [4-NH_2-2-Me(Q)H][VO(ceida)(H_2O)]3H_2O.

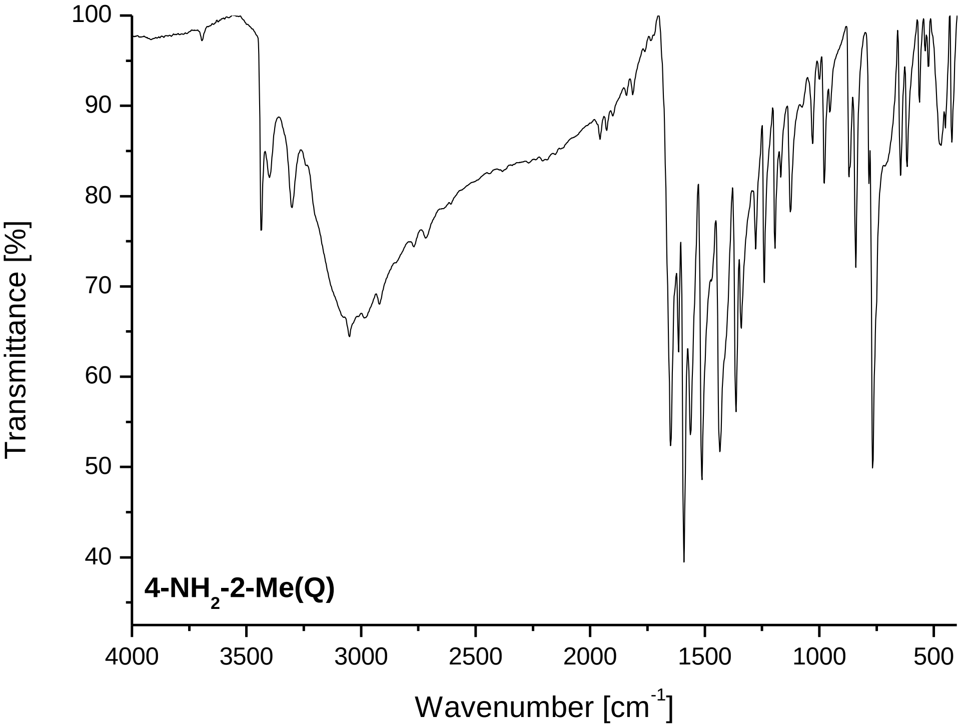

Fig. 3S. The IR spectrum of 4-NH_2-2-Me(Q).

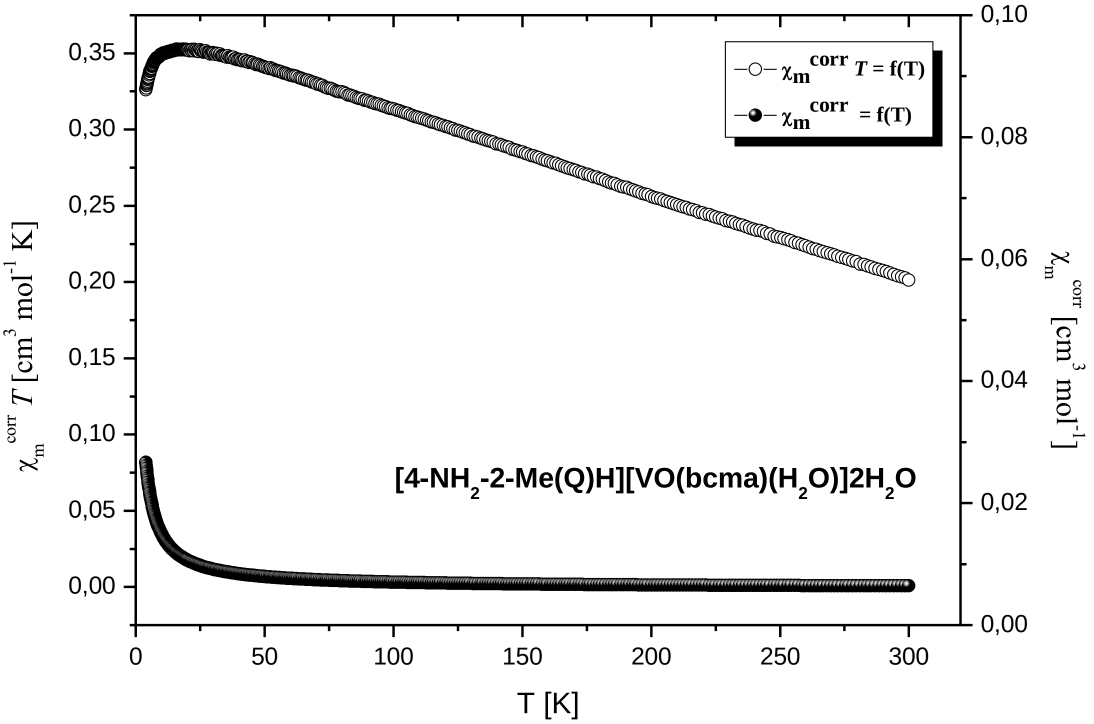

Fig. 4S. The temperature dependence of experimental $\chi_m T$ and χ_m (χ_m per one V(IV) ion) for [4-NH_2-2-Me(Q)H][VO(bcma)(H_2O)]2H_2O.

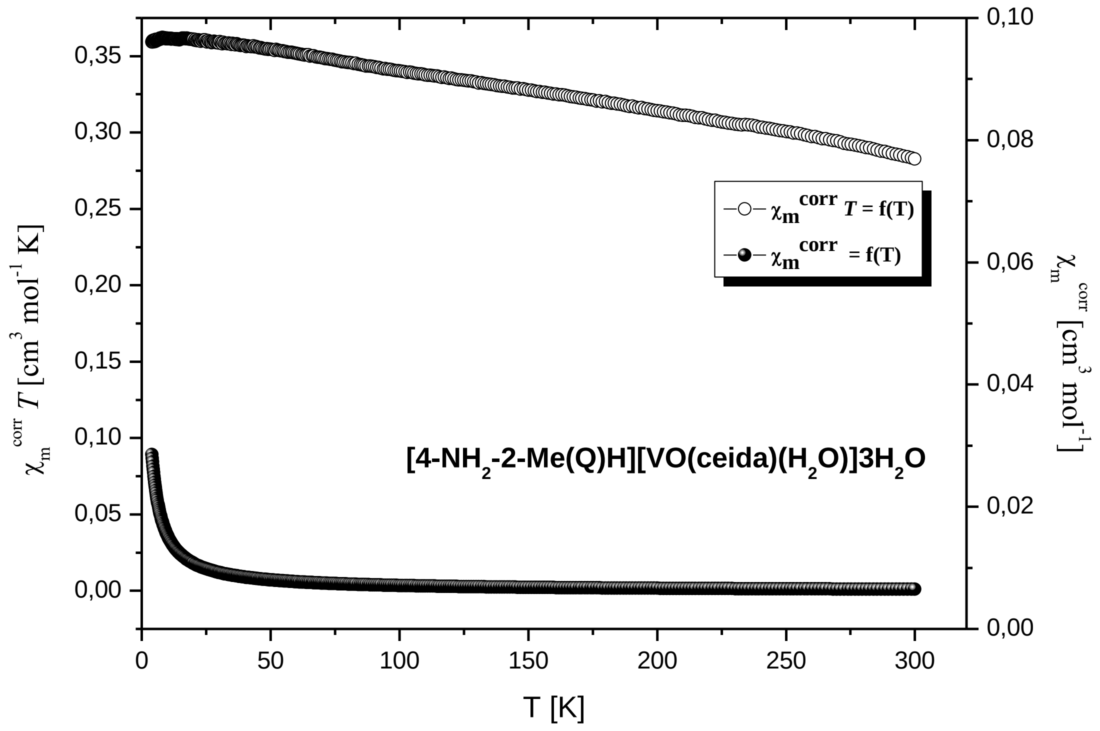

Fig. 5S. The temperature dependence of experimental $\chi_m T$ and χ_m (χ_m per one V(IV) ion) for [4-NH_2-2-Me(Q)H][VO(ceida)(H_2O)]3H_2O.

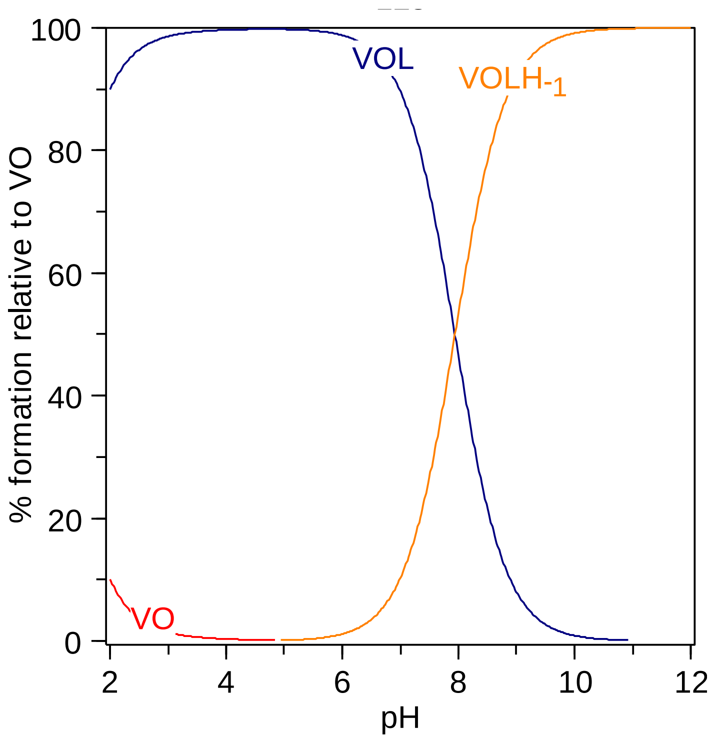

Fig. 6S. Concentration distribution curves of the species presented in the [4-NH_2-2-Me(Q)H]-[VO(bcma)(H_2O)]2H_2O solution as a function of the pH calculated based on the stability constants listed in Table 1.

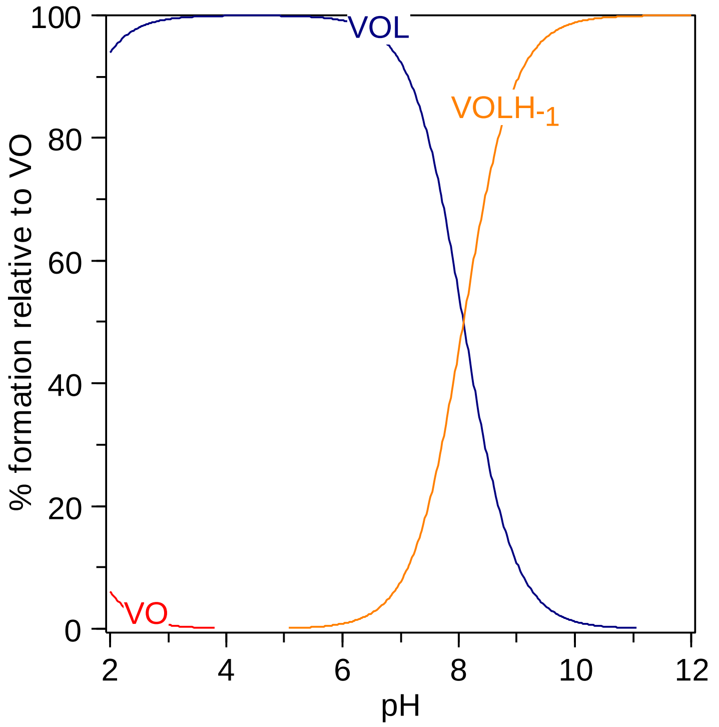

Fig. 7S. Concentration distribution curves of the species presented in the [4-NH_2-2-Me(Q)H]$_2$[VO(pmida)]3H_2O solution as a function of the pH calculated based on the stability constants listed in Table 1.

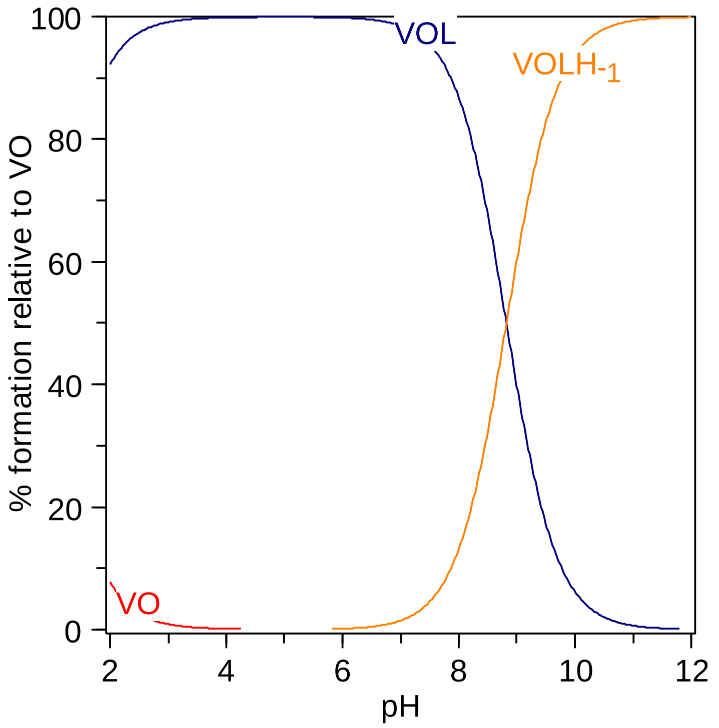

Fig. 8S. Concentration distribution curves of the species presented in the [4-NH_2-2-Me(Q)H][VO(ceida)(H_2O)]3H_2O solution as a function of the pH calculated based on the stability constants listed in Table 1.
